# Improving Drivers’ Hazard Perception and Performance Using a Less Visually-Demanding Interface

**DOI:** 10.3389/fpsyg.2020.02216

**Published:** 2020-09-11

**Authors:** Guy Cohen-Lazry, Avinoam Borowsky

**Affiliations:** Human Performance Evaluation Lab, Industrial Engineering and Management Department, Ben-Gurion University of the Negev, Beer-Sheva, Israel

**Keywords:** hazard perception, in-vehicle interfaces, interface design, multi-touch interface, mental workload, driver distraction, eye movements

## Abstract

In-vehicle devices and infotainment systems occasionally lead to driver distraction, and as a result, increase the risk of missing on-road information. In the current study, a novel multi-touch interface for an in-vehicle infotainment system was evaluated, which potentially requires less visual attention and thus may reduce distraction and increase safety. The interface was compared with a functionally similar control interface in terms of hazard perception metrics and mental workload. Twenty-two participants drove a simulated route once with each system. During each drive, which included eight potentially-hazardous scenarios, participants were instructed to interact with one of the in-vehicle interfaces to perform phone calls or to navigate to specified destinations. Eye-gaze data were collected throughout the drive to evaluate whether participants detected the hazards while interacting with the in-vehicle interface, how much time they needed to identify them, and for how long they engaged with the secondary task. Additionally, after each drive, participants completed a NASA R-TLX questionnaire to evaluate their subjective workload during their engagement with the secondary tasks. Participants using the multi-touch interface needed less time to complete each secondary task and were quicker at identifying potential hazards around them. However, the probability of detecting hazards was similar for both interfaces. Finally, when using the multi-touch interface, participants reported lower subjective workload. The use of a multi-touch interface was found to improve drivers’ performance in terms of identifying hazards quicker than the control condition. The road safety and driver distraction implications of this novel interface are discussed.

## Introduction

While driving, drivers occasionally engage with secondary tasks and become distracted. These tasks may be activities that relate to safety or performance, like using navigational aids (driving-related activities; Pfleging and Schmidt, 2015), or activities that are not related to driving, like phone conversations or radio tuning (non-driving-related activities; Pfleging and Schmidt, 2015). In the United States alone, nine people are killed daily in crashes related to driver distraction, and more than a 1,000 are injured (National Center for Statistics and Analysis, 2017). The most significant negative effect on drivers’ performance is caused by distractions that are both visually and manually demanding (Klauer et al., 2006; Vegega et al., 2013). The diversion of drivers’ gaze away from the forward roadway to the in-vehicle device “affects the degree to which drivers are able to perform primary driving tasks, such as event or object detection, and maintain vehicle control” (Vegega et al. 2013, p. 21).

The visual-manual driver distraction guidelines (National Highway Traffic Safety Administration, 2012), adopted and applied by most manufacturers, suggest that any visual-manual task that may be performed on a system, should be designed in such a way that it “can be completed by the driver while driving with glances away from the roadway of 2 s or less and a cumulative time spent glancing away from the roadway of 12 s or less” (National Highway Traffic Safety Administration, 2012, p. 10). One way to follow these guidelines is to use speech-based interfaces. Studies have shown that, for performing certain tasks, speech-based interfaces improve drivers’ performance in aspects such as lateral positioning (Itoh et al., 2004), speed management (Gärtner et al., 2001), and hazard detection (Ranney et al., 2002). Also, speech-based interfaces reduce the time required to complete tasks and drivers’ subjective workload (Itoh et al., 2004). Nevertheless, other studies have shown contradictory results. Yager (2013), for example, has tested drivers’ distraction by asking drivers to engage in secondary tasks and to respond to occasional illuminating lights. Yager has shown that, even though using speech-based interfaces reduce drivers’ reaction times to the illuminating light compared with manual-interfaces, they still react slower than drivers who do not engage in a data-entry task at all. In another study (Lee et al., 2001), the use of speech-based interfaces caused a 30% increase in drivers’ reaction times to periodic braking of a lead vehicle and introduced a higher workload. In a study regarding cognitive distraction in driving (Strayer et al., 2014), the authors have found that the cognitive demands of speech-based interfaces pose a significant threat to traffic safety, when used for specific tasks such as texting and e-mailing.

In the current research, a novel approach is taken to reduce driver distraction when using an in-vehicle device. A new touch-based interface is evaluated [hereafter multi-touch interface (MTI)], which does not require drivers to gaze toward the screen and thus potentially reduces drivers’ distraction. The MTI is designed as such that, in order to use any command, the driver places three fingers anywhere on the screen, and the system detects their absolute and relative locations and adapts to them. Then, by removing two fingers off the screen, the driver initiates one of three menus (functions) that is uniquely assigned to each finger. The menus, starting from the left-most finger, are a radio menu, a phone menu and a navigation menu. When a particular menu is selected, the driver can slide her finger either up, down, left, or right to select one of four predetermined selections from a star-like menu (i.e., favorites). Each phase is accompanied by an appropriate display in case the driver wishes to verify her actions visually. The fact that the MTI identifies the triple-touch wherever the driver places her fingers reduces the driver’s need to gaze at the screen to search for specific touch-buttons spatially. This feature addresses a significant disadvantage of other touch-based interfaces, which require users to make almost the same number of glances toward them as tactile interfaces to perform tasks (Bach et al., 2008).

A driving simulator study was conducted to compare the MTI with a typical in-vehicle interface [hereafter control interface (CI)] to test the hypothesis that the MTI will help drivers to complete a predefined secondary task quicker than the CI and that the MTI will lead to better hazard perception performance than the CI. The CI that was chosen for this study was a popular infotainment application that was downloaded from Google Play over half a million times and included both a visual-manual and speech-based interfaces. Three relevant measures were chosen to compare the interfaces. First, the time drivers needed to complete a task was recorded, since minimizing the secondary task’s duration is an effective method for reducing driver distraction (National Highway Traffic Safety Administration, 2012) and increasing safety. For the second metric, an eye-tracker was used to measure hazard perception, a measure, which is highly correlated with traffic safety (Horswill and McKenna, 2004; Horswill et al., 2015). Third, whenever a hazard was identified, we measured the time participants needed to identify it, as another indication of hazard perception quality. Finally, the NASA R-TLX was used to test whether the fact that the MTI requires less visual attention also reduces drivers’ workload compared with the CI.

Twenty-two participants were asked to drive two simulated routes, once using each system, during which they were instructed by the experimenter to initiate phone-calls or change the destination in the navigation system. The CI was used either in its visual-manual modality or its speech-based modality. Throughout the drive, various scenarios that required drivers’ attention (not necessarily their action) were initiated, during which drivers’ gaze and task performance were measured. Since the MTI potentially requires fewer number of glances toward the in-vehicle display than the CI, it was expected that:

*Hypothesis* 1: when using the MTI, participants will complete the predefined secondary tasks faster;
*Hypothesis* 2: when using the MTI, participants will be more likely to detect hazards;
*Hypothesis* 3: when using the MTI, participants will identify hazards faster; and
*Hypothesis* 4: when using the MTI, participants will be experience lower levels of workload than the CI.

## Materials and Methods

### Participants

Twenty-two undergraduate students from the Ben-Gurion University (BGU) of the Negev (12 female, ages 21–28 years, *M* = 25.5, *SD* = 2.11) volunteered to a 1-h session, for which they were compensated by course credit. All participants had normal or corrected to normal visual acuity and normal contrast sensitivity. Participants who had glasses were asked to wear contact lenses for the experiment. Participants reported having a valid driver’s license for at least 3 years (*M* = 7.34, *SD* = 2.10), and driving, on average, at least twice a week.

### Apparatus

The experiment was conducted using a medium-fidelity desktop driving simulator. Participants were seated on a gaming seat 1.1 m away from three 24'' LCDs, providing ~90° of horizontal view. The driving simulator was controlled *via* a G27 Logitech steering wheel and a set of pedals. The driving environment was generated using a simulator software provided by Realtime Technologies Inc. (RTI; Royal Oak, MI). The experimental route was a 15-min long drive in an urban environment, in which participants were instructed to keep the right lane whenever possible and drive as they would typically do in similar real-world situations.

**Figure 1:**
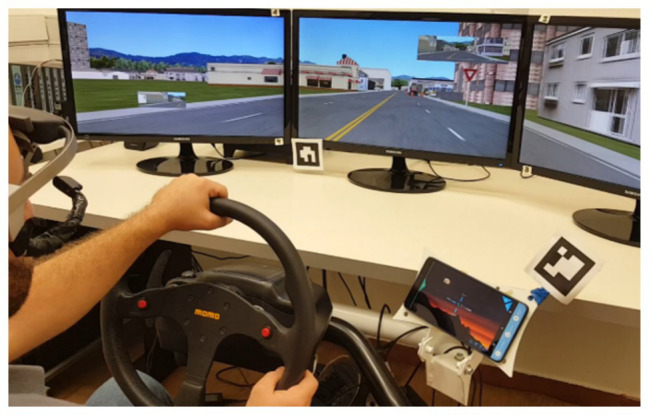
The simulator setup, with the tablet installed to the right of the steering wheel.

Participants’ point of gaze was monitored using a Dikablis light-weight head-mounted eye-tracker (Ergoneers Inc., Manching, Germany). The eye-tracker’s software synchronizes data regarding participants’ gaze with the scene displayed on the simulator screen to provide a measure of where participants’ point of gaze is located at any given moment of the drive. The two types of interfaces (the MTI and the CI) were installed on a 7'' Lenovo tablet. The tablet was positioned to the right of the steering wheel, where an in-vehicle device is commonly located (Figure 1).

### Driving Scenarios

Participants drove two simulated routes, during which they encountered 12 driving scenarios (eight were hazardous scenarios, and four were filler scenarios that did not include any hazard). Participants were also asked to perform tasks using the in-vehicle tablet 12 times during each drive. Eight out of the twelve tasks were given 4 s before a scenario (target or filler). This resulted in eight tasks for which we could measure participants’ hazard perception performance, and four tasks and four scenarios which served as decoys.

The same eight scenarios were used for both drives, but in a randomized order, to allow a direct comparison between the interfaces. Thus, each participant experienced each scenario twice, once while performing a task using the MTI and once while performing the same task using the CI.

### In-Vehicle Tasks

During a drive, participants were verbally instructed by the relevant system to complete two types of tasks. Participants were asked either to make a phone call to one of four pre-programmed numbers (four tasks) or to change the destination in the navigation systems to one of four pre-programmed options (four more tasks). When using the MTI, the entry method was always the multi-touch-based interface. When using the CI, four tasks (Set 1) were completed using a visual-manual (touch) interface, and four tasks (Set 2) were accomplished using a speech-based interface. All tasks using the touch interface required three taps on the screen: one tap to choose the required “app” (i.e., navigation or phone), a second tap to enter the “favorites” screen, and a third tap to choose the requested destination or contact. The speech-based interface required only one click to activate the system’s “listening mode.” Appendix A provides a comprehensive description of the eight scenarios, the type of the associated task and the modality used for that task when using the CI.

### Workload Evaluation

To assess levels of workload, participants filled in the NASA R-TLX. This questionnaire consists of six Likert-style items measuring factors such as “mental demand,” “effort,” and “frustration” on a scale ranging from one (low) to nine (high). Participants filled in the same questionnaire twice, once after using the MTI and once after using the CI.

### Experimental Design

A two (interface type) by eight (scenario) within-subject experiment was designed to minimize the effect of individual differences, and each participant experienced the same eight scenarios using both interfaces. The order of scenarios was randomized so that the two drives did not resemble one another. Additionally, the order of the interfaces that the drivers had to use was counterbalanced between participants. However, for each scenario, the type of task and the modality used (when using the CI) remained the same. For example, during the scenario where a car was pulling into the road from the right shoulder, participants always had to make a phone call, and the modality was always visual-manual (for a complete list, see Appendix A).

### Procedure

Upon arrival at the lab, participants were briefed about the study and were asked to sign an informed consent form. During a 25-min learning session, participants were introduced to both interfaces and to the presets pre-programmed into them (four phone numbers and four destinations). Participants were allowed to practice and engage with both interfaces and to ask questions if they had any. Participants were also introduced to the simulator, where they were allowed to drive for 10 min without the secondary tasks and for 5 min while performing secondary tasks. Then, the two 15-min experimental drives began. Following each drive, participants were asked to fill in the NASA R-TLX questionnaire. After finishing the two driving sessions and filling the questionnaires, participants were debriefed and were allowed to ask questions about the experiment and the study’s goals. The study was reviewed and approved by the internal Human Subjects Research Committee at BGU.

### Dependent Variables

Task duration was calculated as the time interval between the initiation of a pre-recorded auditory request to complete a task (4 s before the beginning of a scenario) and when the task was completed. The end of the task was defined as either releasing the last finger off the screen (MTI), making the last click (CI – visual-manual interface) or finishing the speech command (CI – speech-based interface). A binary variable was used to evaluate hazard identification. Hazard identification was either marked as a success (i.e., participants noticed the hazard in the scenario, “1”) or as a failure (i.e., participants did not notice the hazard in the scenario, “0”). A value of “1” was assigned whenever the participant’s gaze was fixated at the hazard for more than 100 ms (International Organization for Standardization, 2014). Note that since we used an eye-tracking system to evaluate hazard identification, it can only account for identification using the central vision. It may well be that drivers were able to discern the hazard sooner using their peripheral vision, but this would, in any case, require the shift of the central vision system to the location of the hazard to complete its recognition. The time it took participants to identify a hazard was defined as the time interval between the beginning of a scenario (when the hazard instigator became visible) and the participant’s first glance towards it.

### Analysis

Analyses were all conducted using SPSS version 23 (IBM Corp., 2015). A repeated measures mixed-model regression was used, using two independent variables as fixed effects: interface type (MTI or CI) and task type (navigation or phone). Two variables (participants and scenario number) were also included in the models as random effects. The interactions between the variables were not relevant for the current study and were left out of the models. Furthermore, since the MTI was used using only one modality, and the CI was used using two different modalities, the analyses had to be separated. Thus, in order to compare between the two types of interfaces in terms of the various dependent measures, each CI modality (visual-manual or speech-based) was compared to the MTI using a separate regression model within the generalized linear mixed model (GLMM). These two separate regression models were applied once for the secondary task duration (log-linear regression), once for hazard identification (logistic regression) and once for the hazard identification time (log-linear regression).

Each regression model included task type and interface type as fixed effects and participants and scenarios as random effects. All second-order interactions were included in the models. Overall, six different models were used in the analyses (Appendix B provides an extended description of the analyzed models). The significance level was set to *α* = 0.05. Final models were achieved using a backward elimination procedure, and *post hoc* pairwise comparisons were corrected for multiple comparisons using the sequential Bonferroni procedure.

## Results

### Task Duration

**Figure 2:**
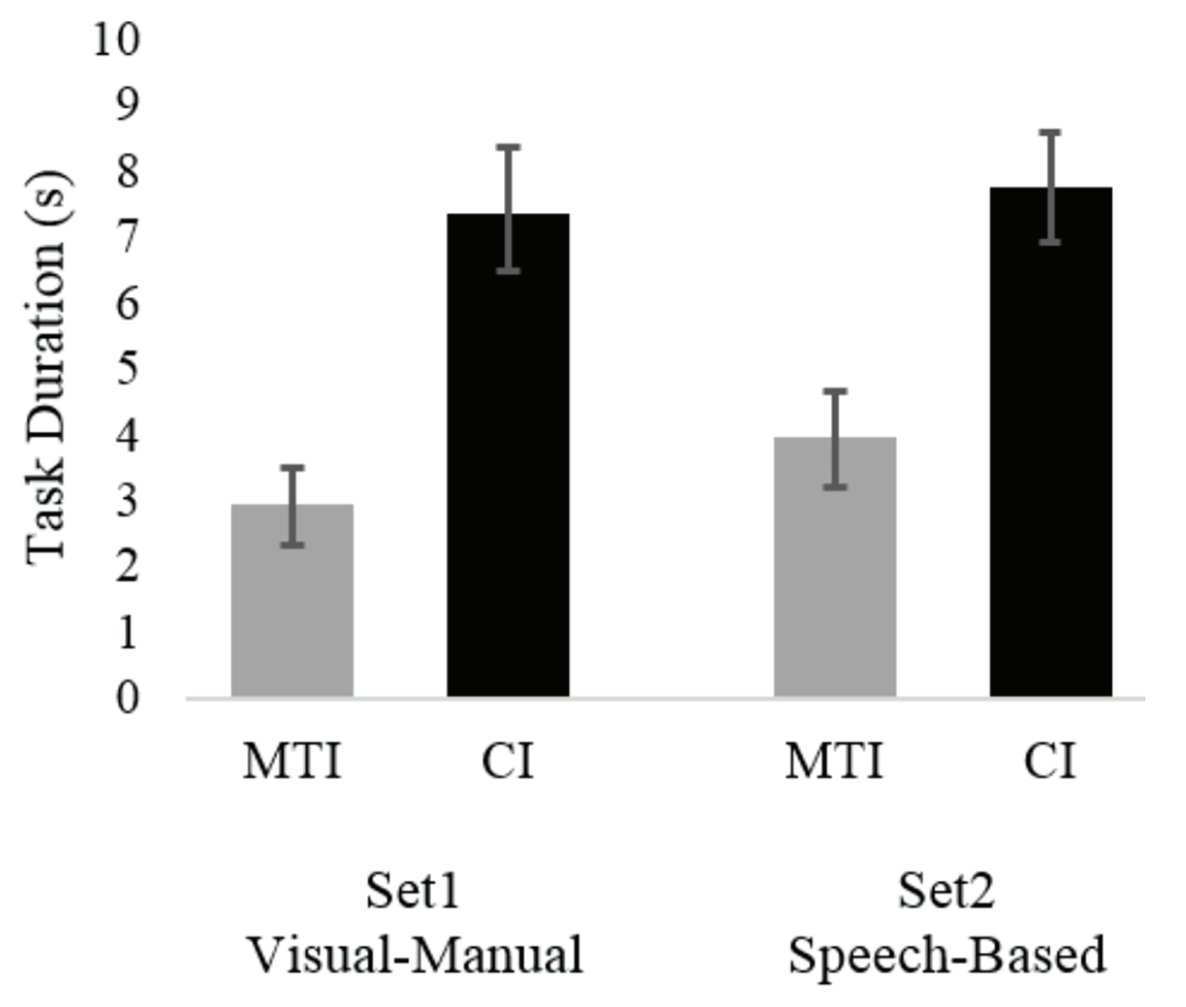
Average task completion duration for the two interfaces and the two scenario sets. Bars represent standard error.

Two linear regression models included a log transformation of the secondary task’s duration as the dependent variable. With regard to the visual-manual modality (Set 1), the final model supported Hypothesis 1 and revealed that the interface type’s main effect was significant [*F*(1, 96) = 44.6, *p* < 0.01], as participants were faster to complete the secondary task when using the MTI (*M* = 2.95 s, *SD* = 2.69) than when using the CI (*M* = 7.34 s, *SD* = 4.14). Task type [*F*(1, 96) = 0.63, *p* < n.s], scenario [*F*(2, 96) = 0.02, *p* < n.s], and participant [*F*(20, 96) = 1.56, *p* < n.s] were all insignificant. Similarly, with regard to the speech-based modality (Set 2), Hypothesis 1 was again supported as it was found that interface type had a significant effect on secondary task’s duration [*F*(1, 87) = 29.8, *p* < 0.01], with participants performing the task faster when using the MTI (*M* = 3.97 s, *SD* = 2.98) than when using the CI (*M* = 7.74 s, *SD* = 3.96). Task type [*F*(1, 87) = 3.55, *p* < n.s], scenario [*F*(2, 87) = 1.12, *p* < n.s], and participant [*F*(20, 87) = 1.11, *p* < n.s] were all insignificant. Means task durations are presented in Figure 2. The averages presented in this figure and every other figure in this paper are based on raw data means and not on the model estimates.

### Hazard Identification Probability

**Figure 3:**
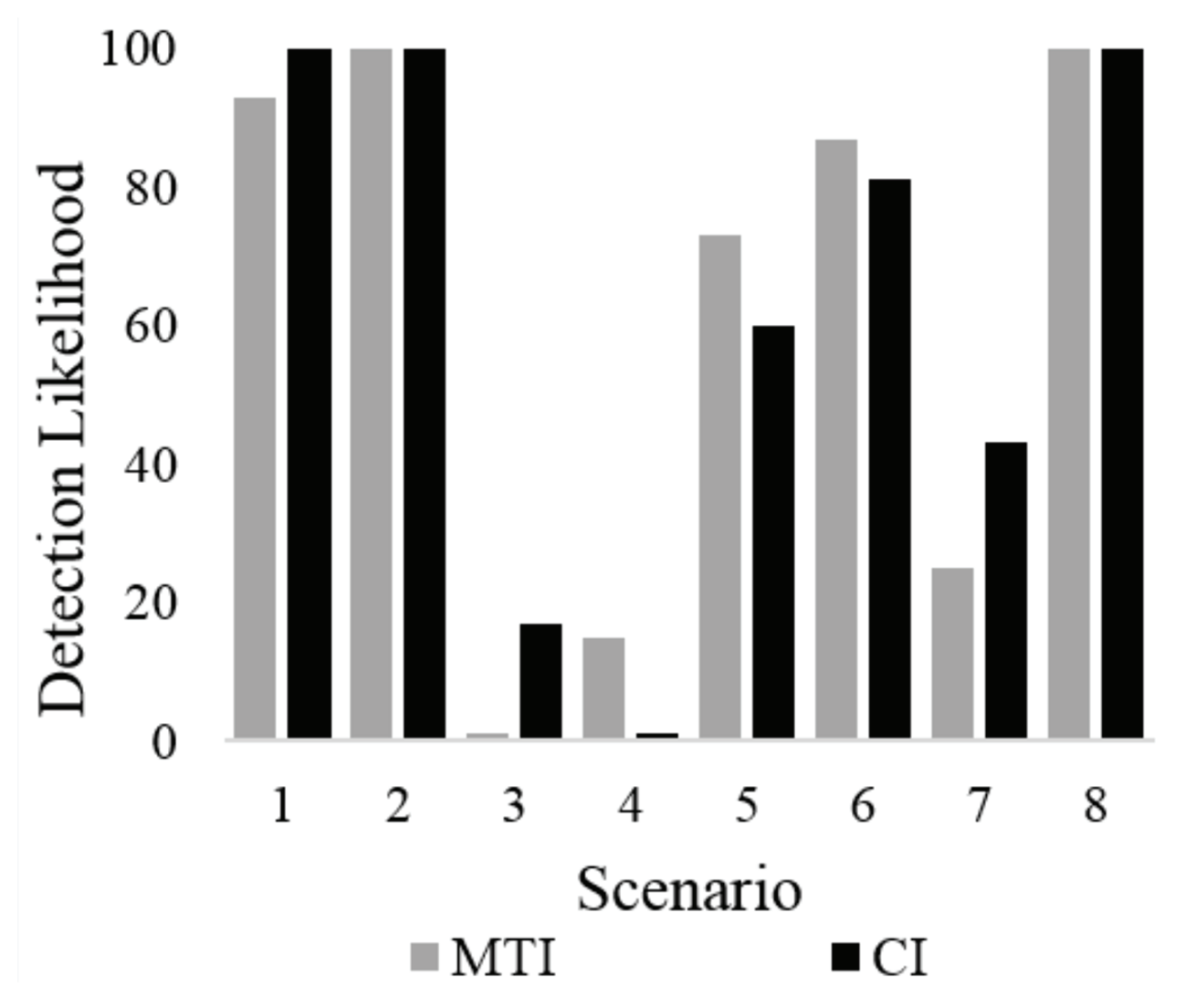
The probability of a participant in either group to detect a hazard, presented per the eight different scenarios.

Two logistic regression models included hazard identification as the dependent variable. With regard to the visual-manual modality, the final model of the first logistic regression did not support Hypothesis 2, as it revealed that interface type did not significantly affect participants’ probability of detecting a hazard, *χ*^2^(1) = 0.19, *p* = n.s; participants identified 61% of all hazards when using the MTI and 57% of all hazards when using the CI. Among all other variables, scenario was the only significant variable, *χ*^2^(3) = 10.93, *p* < 0.01, whereas task type *χ*^2^(1) = 0.01, *p* < n.s and participant *χ*^2^(20) = 4.51, *p* < n.s were both insignificant. Similarly, with regard to the speech-based interface, Hypothesis 2 was again not supported since the final model of the second logistic regression revealed that interface type did not significantly affect participants’ probability of detecting a hazard, *χ*^2^(1) = 0.57, *p* = n.s; participants identified 72% of all hazards when using the MTI and 70% of all hazards when using the CI. Again, among all other variables, scenario was the only significant variable, *χ*^2^(3) = 14.11, *p* < 0.01 whereas task type *χ*^2^(1) = 4.12, *p* < n.s and participant *χ*^2^(20) = 7.95, *p* < n.s were both insignificant. Hazard detection rates for the different scenarios are presented in Figure 3.

### Hazard Identification Time

**Figure 4:**
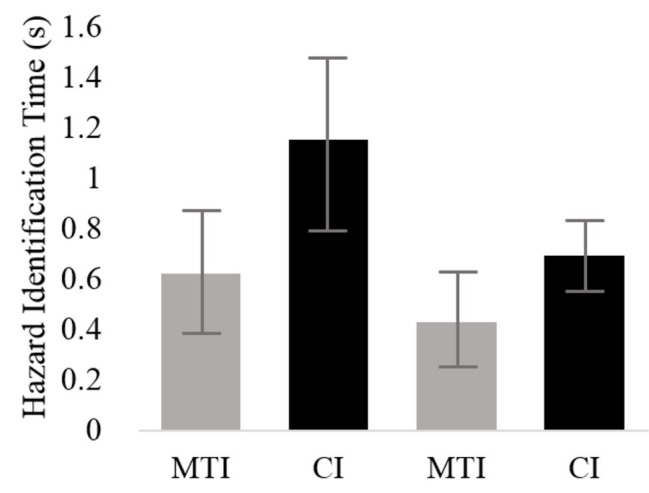
Average hazard-identification durations for the two interfaces and the two scenario sets. Bars represent standard error.

Two linear regression models included a log transformation of hazard identification time as the dependent variable. With regard to the manual-visual modality, the final model of the first linear regression supported Hypothesis 3 and revealed that the effect of interface type was significant [*F*(1, 91) = 47.16, *p* < 0.01], with participants identifying the hazards quicker when using the MTI (*M* = 0.65 s, *SD* = 1.70) than when using the CI (*M* = 1.20 s, *SD* = 2.63). Task type [*F*(1, 91) = 0.62, *p* < n.s], scenario [*F*(2, 91) = 0.74, *p* < n.s], and participant [*F*(20, 91) = 1.52, *p* < n.s] were all insignificant. Similar results, supporting Hypothesis 3, were found for the second linear regression model with regard to the speech-based modality such that interface type had a significant effect on hazard identification time [*F*(1, 93) = 31.93, *p* < 0.05], with participants identifying the hazards quicker when using the MTI (*M* = 0.45 s, *SD* = 1.27) than when using the CI (*M* = 0.72 s, *SD* = 0.75). Task type [*F*(1, 93) = 3.77, *p* < n.s], scenario [*F*(2, 93) = 1.81, *p* < n.s], and participant [*F*(20, 93) = 2.05, *p* < n.s] were all insignificant. Estimated means of the hazard identification times are presented in Figure 4.

### NASA R-TLX

**Figure 5:**
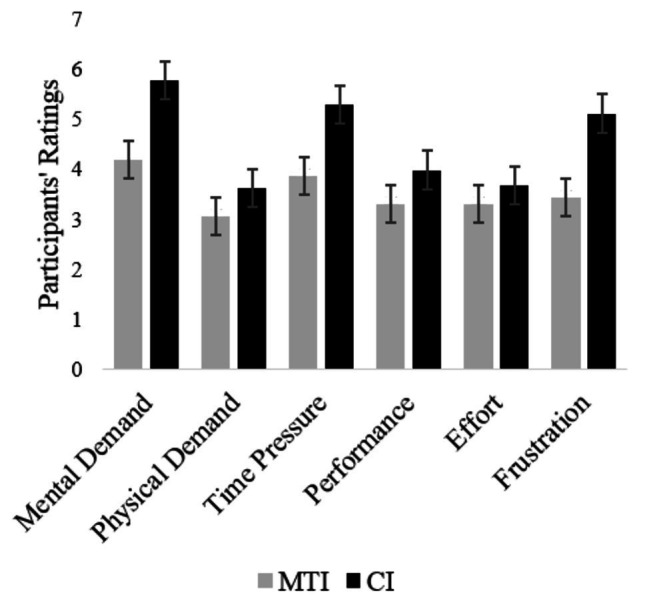
Average NASA R-TLX ratings for the six factors, comparing the multi-touch interface (MTI) and the control interface (CI). Bars represent standard error.

The fourth item in the NASA R-TLX, regarding task performance, is rated on an inverse scale (1 – high performance, 9 – low performance) and was inversed before data analysis. To compare the workload that participants experienced while using each interface, a repeated-measures analysis of variance (ANOVA) was conducted with R-TLX ratings as the dependent variable and interface type as a within-subject fixed factor. As hypothesized (Hypothesis 4), there was a significant effect of interface type on participants’ workload ratings, *F*(1, 87) = 16.64, *p* < 0.01. The difference between the questionnaire’s items was insignificant, *F*(5, 87) = 2.04, *p* < n.s. The NASA R-TLX ratings for each item, presented in Figure 5, show that the MTI scored lower than the CI across all effort and pressure factors, indicating lower workload. When asked about their task performance using each one of the in-vehicle interfaces (fourth item on the NASA R-TLX), participants rated the CI significantly higher, meaning they thought that their performance was better when interacting with it than when interacting with the MTI (Figure 5). This result is intriguing since the aforementioned objective measures have indicated better performance when using the MTI.

## Discussion

This study was aimed at evaluating a novel concept of interfaces for in-vehicle devices that target the reduction of driver distraction and the increase of safety. The interface was compared with a control interface allowing both visual-manual and speech-based interactions. Since all findings were similar for both types of input modalities, from hereon, we will disregard this difference between modalities and only discuss the differences between the two systems. Results point to a significant improvement in three distraction-related measures. First, in-line with Hypothesis 1, when using the MTI, the time participants needed to complete each task was significantly reduced. One possible explanation for the longer time participants needed to complete tasks using the CI is that, to operate it, participants had to visually locate and aim their finger at the right touch-button three times. The MTI, on the other hand, could be operated anywhere on the screen, without glancing towards it even once. Second, as predicted in Hypothesis 3, participants identified hazards faster when using the MTI. Finally, as expected in Hypothesis 4, the workload participants reported was also significantly reduced when the MTI was used. The decrease in task duration time is an essential aspect in designing in-vehicle interfaces, and it has been shown that the longer people glance away from the road to perform a secondary task, the likelihood of a crash increases (e.g., Simons-Morton et al., 2014). Burns et al. (2010) have discussed the negative effect of long task-durations and have suggested that a key manner in which this risk could be decreased is by designing interfaces that support quicker performance. The results of the current study are consistent with their claim, showing that indeed a shorter task-duration may lead to an increase in safety. Despite these essential improvements, Hypothesis 2 was disconfirmed as we did not find any advantage for the MTI concerning drivers’ probability of identifying a hazard. One possible explanation regards the overall difficulty of identifying hazards in the various scenarios.

By examining the identification probabilities in Figure 3, it is evident that in six out of the eight scenarios, the probability of identifying a hazard was either very high or very low. This suggests that, in most cases, identifying a hazard was either very easy (ceiling effect) or very difficult (floor effect) when using both interfaces, thus reducing the possibility of revealing significant differences between them. Nevertheless, despite this lack of difference between the interfaces, when using the MTI, participants were faster at identifying hazards than when they were using the CI. Possibly, this difference is rooted in the experimental design.

Throughout the experiment, the time between the requirement to complete a task and the initiation of a scenario was fixed at 4 s. Additionally, as seen in Figure 2, participants using the MTI needed, on average, less than 4 s to complete a task, whereas participants using the CI needed more than 7 s. Thus, it seems that, on average, participants using the MTI completed their tasks before the initiation of the hazardous scenario. Hence, throughout the entire duration of the scenario, participants were not distracted by a secondary task and could divert all their attention to the road. Conversely, participants using the CI were still engaged in performing the secondary task for a few more seconds when the scenario started. Nevertheless, they were still able to identify the hazard before the end of the hazardous scenario. These task duration differences between the groups explain why the hazard identification times were shorter for the MTI even though the identification probabilities were similar for both interfaces. Participants using the MTI had their full attention allocated to monitoring the environment throughout the entire scenario, whereas participants using the CI had to divide their attention between the secondary task and the road environment, at least for a few seconds. Still, even though participants who were using the CI began monitoring the environment later, the relatively long duration of the scenarios (~10 s) and the aforementioned ceiling and floor effects allowed them to identify the hazards at a similar likelihood to that of participants who were using the MTI. This might explain the similarities in identification probabilities alongside with the differing identification times.

This analysis of task duration may also put into perspective the results regarding the hazard identification times. Since drivers using the MTI completed their tasks before the initiation of the scenario, they did not, in fact, identify hazards while performing secondary tasks. Therefore, their superiority in identifying hazards faster than participants using the CI may be an effect of performing a single task and not two tasks simultaneously, which is a well-known advantage in hazard perception tasks (e.g., Burge and Chaparro, 2018). Further research is required to determine whether the MTI also reduces hazard identification times during the performance of secondary tasks. Nevertheless, even if the faster hazard identification times are the result of performing just one task, this result still denotes an advantage in favor of the MTI.

This study’s results suggest an advantage for a multi-touch-only interface over common tactile interfaces. However, several limitations have to be acknowledged. First, the sample size and its homogeneity (undergraduate students) limit the results’ generalizability. Second, using the touch-only interface may pose requirements (e.g., a certain level of dexterity) or have implications that were not studied here. Jin et al. (2007), for example, have shown that touch interfaces have to be designed differently when designing for the elderly. This aspect of the interface was not examined in this study and should be a part of future studies. Third, while this study focused on the real-time hazard perception-related effects, the introduction of a new interface probably has long-term effects as well. Specifically, future research should examine people’s attitudes towards the interface (e.g., their trust or annoyance with it), and whether they find it useful. Fourth, due to technical limitations, the order of scenarios was not randomized between participants. Although this could have led to a learning effect, an examination of Figure 3 suggests that even if such an effect existed, it affected both groups similarly, as indicated by their similar detection rates throughout all scenarios. Finally, while the study compared the MTI with a control interface, it did not use a no-task reference condition as a baseline for drivers’ non-distracted performance. Therefore, although the MTI showed significant advantages when compared to the CI, it is not possible to tell how distracting the system is when compared to driving without a non-driving-related secondary task.

This study, thus, has shown that the MTI, which is based on a non-visual MTI, has two advantages over a representative in-vehicle touchscreen interface and a speech-based interface. Participants using MTI needed less time to complete phone and navigation tasks and also experienced a lower workload. These two variables are closely related to the concept of hazard perception and thus suggest a significant potential for systems such as the MTI in reducing driver distraction and enhancing safety (e.g., Burns et al., 2010) An analysis of the results regarding hazard identification time and hazard identification probabilities pointed to issues in the experimental design that provide possible alternative explanations for some of the results. Thus, concerning these two variables, we are currently unable to determine whether the MTI does or does not have an advantage over the CI. Further studies will be designed to allow an exploration of these other variables using different scenario designs and timings.

## Data Availability Statement

The raw data supporting the conclusions of this article will be made available by the authors, without undue reservation.

## Ethics Statement

The studies involving human participants were reviewed and approved by the Human Subjects Research Committee of the Ben-Gurion University. The patients/participants provided their written informed consent to participate in this study.

## Author Contributions

All authors listed have made a substantial, direct and intellectual contribution to the work, and approved it for publication.

### Conflict of Interest

The authors declare that the research was conducted in the absence of any commercial or financial relationships that could be construed as a potential conflict of interest.

## Figures and Tables

**Table A1:** Driving scenarios, tasks, and input modalities.

	Scenario	Type of task	Experimental modality	Control system modality
Set 1	**A bus station close to the road (scenario 7)** A detection was defined as looking behind the bus station to make sure no pedestrians walk there	NAV	MT	VM
	**A tree hiding the sidewalk during a turn (scenario 6)** A detection was defined as looking for a hidden pedestrian behind the tree	NAV	MT	VM
	**A construction site (scenario 4)** A detection was defined as gazing at the site to make sure no workers barge into the road	PHC	MT	VM
	**A car pulling into the road (scenario 2)** A detection was defined as observing the car as it pulled out and into the road	PHC	MT	VM
Set 2	**A car unable to maintain lane and speed (scenario 8)** A detection was defined as making more than one observation of that vehicle	NAV	MT	SB
	**A playground near the road (scenario 1)** A detection was defined as gazing at the site to make sure no workers barge into the road	NAV	MT	SB
	**A stopped truck on the right lane (scenario 5)** A detection was defined as observing the car as the driver passed by it	PHC	MT	SB
	**A phone booth near the road (scenario 3)** A detection was defined as looking for a pedestrian to walk from within or behind the booth	PHC	MT	SB

VM, visual manual; MT, multi-touch; SB, speech-based; NAV, navigation; PHC, phone call.

**Table A2:** A summary of the regression models used for the analyses.

		Dependent variable		
		Task completion time	Hazard-identification	Hazard-identification time
Control system interface	Visual-manual (Set 1 scenarios)	Regression model 1	Logistic regression model 1	Regression model 3
	Speech-based (Set 2 scenarios)	Regression model 2	Logistic regression model 2	Regression model 4

## Appendix A

Out of the eight hazardous scenarios, four included a materialized hazard, like a car on the shoulders pulling out into the road or a car that fails to maintain its lane position or speed properly. The four other scenarios included latent hazards, like workers standing in a construction area next to the road (that could jump into the participant’s lane at any moment) or a playground next to the road (from where a child could run into the road). Table A1 describes the eight scenarios.

## Appendix B

Two different general linear mixed models were used to analyze each dependent variable. One regression was used to model the differences between the experimental system and the control system when using the visual-manual modality in both systems, and the other was used to model the differences between the systems when using the speech-based modality in the control system and the multi-touch modality in the experimental system. Notably, participants in the experimental system used only the visual-manual modality. These two separate regression models were applied once for the secondary task completion time (log-linear regression) dependent variable, once for hazard identification (logistic regression) dependent variable, and once for the hazard identification time (log-linear regression) dependent variable. Overall, six models were analyzed, as shown in Table A2.
